# Association between vitamin D deficiency and the 8-year risk of incident depression in patients with rheumatoid arthritis: a cohort study

**DOI:** 10.3389/fnut.2026.1845922

**Published:** 2026-05-21

**Authors:** Kuo-Mao Lan, Ying-Jen Chang

**Affiliations:** 1 Department of Anesthesiology, Chi Mei Hospital, Liouying, Tainan City, Taiwan; 2 Department of Anesthesiology, Chi Mei Medical Center, Tainan City, Taiwan; 3 Department of Recreation and Health-Care Management, College of Recreation and Health Management, Chia Nan University of Pharmacy and Science, Tainan City, Taiwan

**Keywords:** depression, propensity score matching in cohort study, rheumatoid arthritis, TriNetX, vitamin D deficiency

## Abstract

**Background:**

Depression is common among patients with rheumatoid arthritis (RA) and is associated with adverse clinical outcomes. Vitamin D deficiency (VDD) is also prevalent in patients with RA and has been linked to depressive symptoms in cross-sectional studies; however, longitudinal data on its association with incident depression are limited.

**Methods:**

We conducted a retrospective cohort study using the TriNetX Global Collaborative Network (2010–2024). Adults with RA and serum 25-hydroxyvitamin D levels were identified and classified as having VDD (<20 ng/mL) or sufficient vitamin D levels (≥30 ng/mL). After 1:1 propensity score matching for demographics, comorbidities, medications, and laboratory variables, 18,339 patients were included in each group. A 180-day landmark period was applied to strengthen the temporal ordering. The primary outcome was the incidence of depression. The secondary outcomes included antidepressant use, suicide-related events, and all-cause mortality. Negative and positive control outcomes were also examined.

**Results:**

Compared with sufficient vitamin D levels, VDD was associated with a higher risk of incident depression (HR 1.64, 95% CI 1.50–1.78; *p* < 0.001). Similar patterns were observed for antidepressant use (HR 1.46, *p* < 0.001), suicide-related events (HR 1.62, *p* = 0.045), and all-cause mortality (HR 1.40, *p* < 0.001). The negative control outcome was not significantly associated with VDD, whereas the positive control outcome showed the expected association. The findings were generally consistent across sensitivity, subgroup (sex-based), and exposure-gradient analyses.

**Conclusion:**

Among patients with RA, VDD was associated with a higher long-term risk of incident depression. These findings support a possible association between vitamin D status and depression risk in this population; however, a causal inference cannot be established from this observational study.

## 1 Introduction

Rheumatoid arthritis (RA) is a chronic systemic autoimmune disease characterized by persistent joint inflammation, progressive functional disability, and diminished quality of life (1, 2). Beyond its musculoskeletal manifestations, RA is increasingly being recognized as a condition associated with a substantial psychiatric burden, with depression being one of the most prevalent comorbidities. Epidemiological evidence indicates that the prevalence of depression in patients with RA is approximately two to three times higher than that in the general population, and the presence of comorbid depression has been linked to poorer treatment adherence, increased disability, and elevated mortality risk (3, 4). Despite this well-documented burden, modifiable risk factors for incident depression in the RA population remain incompletely characterized.

Vitamin D deficiency (VDD) is highly prevalent among patients with RA, with reported rates ranging from 30% to over 60% (5), depending on the population studied and the threshold used for defining deficiency. Several biological mechanisms have been proposed to explain a potential link between low vitamin D levels and depression, including the role of vitamin D in serotonin synthesis, neuroinflammatory regulation, and hypothalamic-pituitary-adrenal axis modulation (6, 7). In the general population, observational studies and meta-analyses have consistently demonstrated an inverse association between serum 25-hydroxyvitamin D levels and the risk of depression (8, 9). However, whether VDD is longitudinally associated with an elevated risk of incident depression specifically in patients with RA has not been investigated. Prior studies examining the relationship between vitamin D status and depression in RA have been limited to cross-sectional designs with small sample sizes, precluding the assessment of temporal directionality (10, 11). Given the dual burden of VDD and depression in RA and the potential clinical implications for the early identification of at-risk patients, clarification of this association in a longitudinal framework is warranted.

Therefore, this study aimed to examine the association between VDD and the long-term risk of incident depression in a large cohort of patients with RA using propensity score matching and a landmark analysis design to strengthen temporal inference.

## 2 Methods

### 2.1 Data sources and exposure definition

This retrospective cohort study was conducted using the TriNetX Global Network, a federated database of de-identified electronic health records. Additional details regarding the data source and coding framework are provided in Supplementary Table 1. This retrospective cohort study used de-identified electronic health record data from the TriNetX Global Collaborative Network. The study protocol was reviewed and approved by the Institutional Review Board of Chi Mei Medical Center, which granted a waiver of informed consent because all data were de-identified and compliant with relevant data protection regulations. All procedures were conducted in accordance with the ethical standards of the institutional research committee and the Declaration of Helsinki.

Patients were identified from the TriNetX Global Collaborative Network between January 1, 2010, and December 31, 2024. Eligible participants were adults aged ≥18 years with a diagnosis of rheumatoid arthritis and at least one measurement of serum 25-hydroxyvitamin D during the study period. Rheumatoid arthritis was defined using the International Classification of Diseases, Tenth Revision (ICD-10) codes for rheumatoid arthritis with rheumatoid factor (M05) or other rheumatoid arthritis (M06). Patients were assigned to the VDD group if their serum 25-hydroxyvitamin D level was < 20 ng/mL, and to the control cohort if their serum 25-hydroxyvitamin D level was ≥30 ng/mL (12). The index date was defined as the date of the qualifying vitamin D measurement. To ensure exposure classification stability, patients in the VDD cohort were excluded if they had any prior vitamin D measurement ≥30 ng/mL within the preceding 3 years. Similarly, patients in the control cohort were excluded if they had any prior vitamin D measurement < 20 ng/mL within the same period.

### 2.2 Exclusion criteria

To ensure incident outcome assessment, patients were excluded if they had any history of depressive disorders, suicidal ideation, suicide attempts, intentional self-harm, or antidepressant use prior to the index date. Additional exclusion criteria included advanced renal disease (i.e., end-stage renal disease, chronic kidney disease stage 4 or 5, or dialysis dependence), bipolar disorder, schizophrenia or other psychotic disorders, and a history of bariatric surgery. Patients with a history of stroke (ischemic or hemorrhagic) within 1 year before the index date were also excluded to reduce potential reverse causation related to post-stroke depression. A landmark analysis design was used to strengthen the temporal ordering between exposure and outcome; therefore, outcomes occurring within the first 180 days after the index date were excluded. Patients were followed from 180 days after the index date until the occurrence of depression, death, or the end of the study period, whichever came first. The codes used for cohort definition, inclusion criteria, exclusion criteria, and outcome definitions are summarized in Supplementary Table 2.

### 2.3 Data collection and propensity score matching

To reduce baseline imbalances between the VDD and control groups, 1:1 propensity score matching was performed using greedy nearest-neighbor matching with a caliper of 0.1 standard deviations of the logit of the propensity score. Matching was based on key baseline variables that could influence both vitamin D status and depression risk, including demographic characteristics (age, sex, and race), major medical comorbidities, RA-related treatment, selected laboratory measures, and indicators of healthcare utilization.

Major comorbidities, such as diabetes mellitus, chronic kidney disease, cerebrovascular disease, thyroid disorders, malnutrition, and anxiety-related disorders, were included to account for overall medical and psychiatric vulnerability. RA-related medications, including corticosteroids, methotrexate, hydroxychloroquine, and tumor necrosis factor inhibitors, were also incorporated to partially reflect disease severity and treatment intensity. In addition, selected laboratory variables, such as albumin, C-reactive protein, hemoglobin A1c, and body mass index, were included to capture nutritional status, systemic inflammation, and metabolic conditions. Detailed definitions of all matching variables are provided in Supplementary Table 2. Covariate balance after matching was assessed using standardized mean differences (SMDs), with an SMD < 0.1 considered indicative of adequate balance.

### 2.4 Primary and secondary outcomes

The primary outcome was incident overall depression, defined as a new diagnosis of depressive episode or recurrent depressive disorder, as identified using the ICD-10 codes F32 and F33. To further characterize the primary outcome, depressive subtypes, including depressive episode and recurrent depressive disorder, were analyzed separately. Secondary outcomes included antidepressant use, suicide-related outcomes (suicidal ideation, suicide attempt, or intentional self-harm), and all-cause mortality. Osteoporosis with pathological fractures was included as a positive control outcome because of its well-established association with VDD.

In addition, two supportive outcomes were evaluated to strengthen the interpretation of the findings. Healthcare visits were assessed to examine whether the VDD group had greater healthcare utilization during the follow-up, which could indicate potential surveillance bias. Subsequent VDD during follow-up was also examined to assess the stability of the initial exposure classification and to confirm whether patients in the VDD group remained more likely than controls to experience subsequent VDD.

### 2.5 Sensitivity analyses

Several sensitivity analyses were performed to examine the robustness of the primary findings and to reduce the influence of residual confounding and misclassification. First, the analysis was restricted to the period from 2018 to 2024 to reflect a more contemporary cohort and to reduce potential heterogeneity related to temporal changes in clinical practice, vitamin D testing, and depression diagnosis. Second, the study population was restricted to patients with RA who also received RA-related drug treatment (Supplementary Table 3) to improve diagnostic specificity and reduce misclassification. Third, only patients with a history of RA of > 1 year before the index date were included to ensure a more stable underlying disease status. Additional sensitivity analyses were performed by excluding patients with other nutritional deficiencies, those with a history of neoplasms, and those with a history of chronic pain or sleep disorders, as these conditions may independently influence both vitamin D status and depression risk. All sensitivity analyses were conducted using the same propensity score matching strategy and statistical approach as in the primary analysis.

### 2.6 Subgroup analyses

Subgroup analyses were performed to evaluate whether the association between VDD and incident depression differed across patient subgroups. Pre-specified subgroup analyses were conducted according to sex (male and female) and age group. Separate propensity score matching and Cox proportional hazards analyses were performed for each subgroup. The interaction effects between vitamin D status and subgroup variables were assessed to determine whether the association differed significantly across subgroups.

### 2.7 Exposure-gradient analysis

To evaluate a potential dose–response relationship between vitamin D status and depression risk, an exposure–gradient analysis was performed. In addition to the primary comparison between VDD (< 20 ng/mL) and sufficient vitamin D levels (≥30 ng/mL), a vitamin D insufficiency group (20–29.9 ng/mL) was identified and compared with the same control group with sufficient vitamin D levels. Propensity score matching was performed separately for the insufficiency and control groups using the same matching variables and methods as in the primary analysis.

### 2.8 Statistical analysis

Associations between vitamin D status and study outcomes were examined using Cox proportional hazards regression, with effect estimates reported as hazard ratios (HRs) and 95% confidence intervals (CIs). Because the main purpose of this study was to investigate etiologic relationships between vitamin D status and subsequent depression, we applied a cause-specific Cox approach rather than a competing-risk model, such as the Fine–Gray subdistribution hazard model, although death may preclude the occurrence of depression during follow-up. This approach is generally considered appropriate when the focus is on the association between exposure and outcome rather than on the direct prediction of cumulative incidence in the presence of competing events.

All analyses were conducted using the available data without imputation for missing values. The proportional hazards assumption was evaluated using Schoenfeld residuals, with a two-sided *P* value >0.05 interpreted as indicating no meaningful departure from the assumption. Cumulative event-free survival was estimated using the Kaplan–Meier method, and differences between groups were assessed using the log-rank test.

To assess the possible impact of residual unmeasured confounding factors, E-values were calculated for the primary outcome. The E-value reflects the minimum magnitude of association that an unmeasured confounder would need to have with both the exposure and outcome, independent of the measured covariates, to account for the observed association. Statistical significance for the primary outcome was defined as a two-sided *P*-value < 0.05. Analyses involving secondary outcomes, control outcomes, sensitivity analyses, and subgroup comparisons were interpreted as exploratory, and no formal correction for multiple tests was applied. Therefore, these results should be interpreted cautiously, as some findings—particularly those with borderline statistical significance—may not remain significant after adjustment for multiple comparisons.

## 3 Results

### 3.1 Patient selection and baseline characteristics

**Figure 1:**
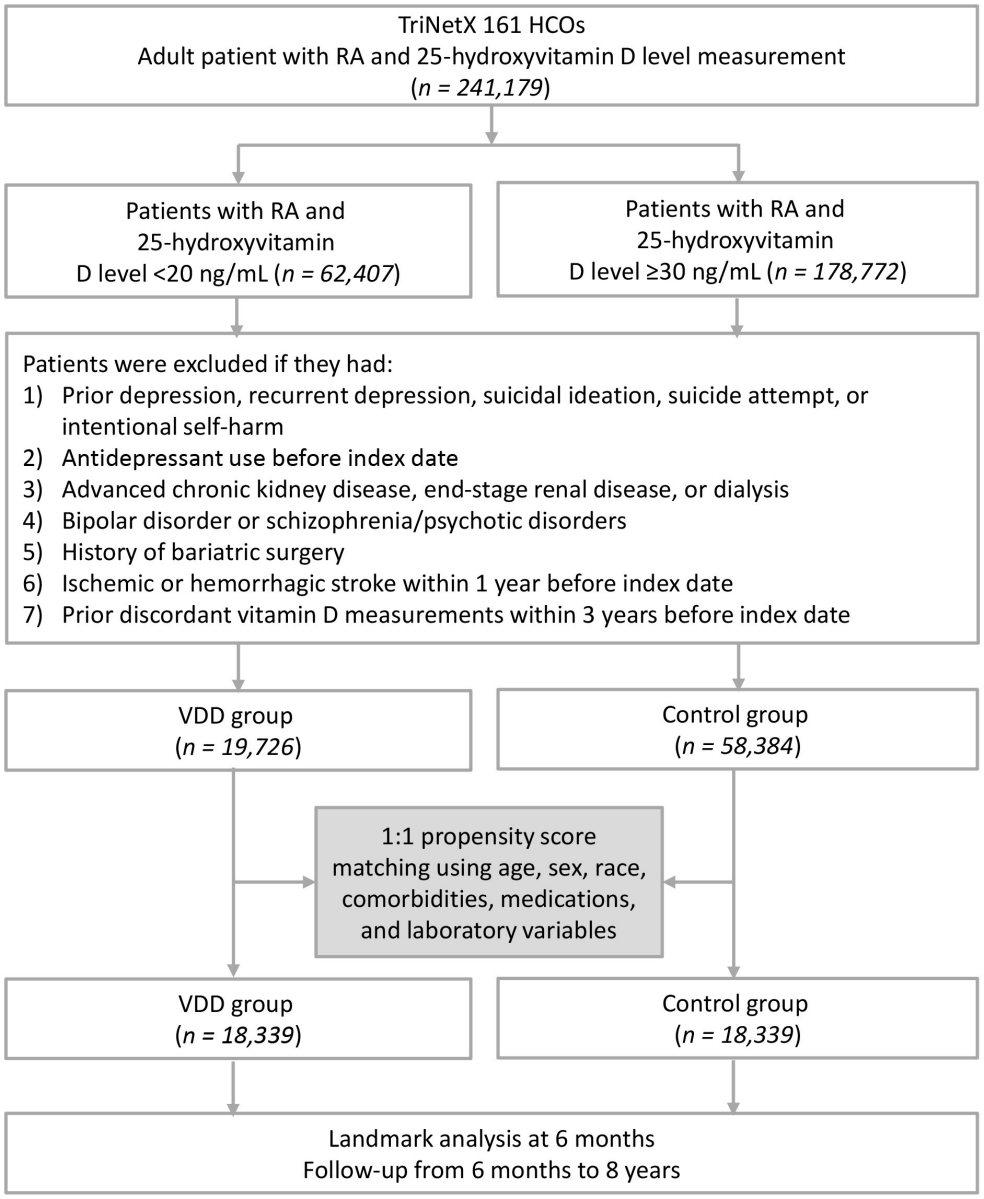
Flowchart of patient selection. HCOs, healthcare organizations; VDD, vitamin D deficiency; CKD, chronic kidney disease; ESRD, end-stage renal disease. RA, rheumatoid arthritis.

After applying the exclusion criteria, 19,726 patients with RA and VDD and 58,384 healthy controls with sufficient vitamin D levels were identified from the TriNetX Global Collaborative Network. Following matching, 18,339 patients remained in each group for the final analysis (Figure 1). Before matching, imbalances were observed between the groups in terms of age (mean 55.6 vs. 62.8 years), race (White: 50.6% vs. 71.4%; Black or African American: 23.2% vs. 10.7%), and several clinical characteristics, such as C-reactive protein ≥10 mg/L (27.7% vs. 18.7%), albumin ≤ 3.5 g/dL (23.5% vs. 16.7%), and nicotine dependence (10.5% vs. 5.6%). After propensity score matching, all standardized mean differences were < 0.1, indicating balance across demographic characteristics, comorbidities, laboratory variables, medications, and healthcare utilization indicators (Table 1). Several laboratory variables showed partial missingness. After propensity score matching, the availability of laboratory data ranged from 30% to 81%, and the proportions of available data were similar between the VDD and control groups (Supplementary Table 4).

**Table 1:** Baseline characteristics of patients with vitamin D deficiency and controls before and after propensity score matching.

Variables	Before matching			After matching		
	VDD group (*n* = 19,726)	Control group (*n* = 58,384)	SMD	VDD group (*n* = 18,339)	Control group (*n* = 18,339)	SMD
Patient characteristics						
Age at index (years)	55.6 ± 16.4	62.8 ± 14.4	0.468	56.8 ± 15.8	56.7 ± 16.0	0.002
BMI ≥30 (kg/m^2^)	7,652 (38.8)	17,613 (30.2)	0.182	6,861 (37.4)	6,810 (37.1)	0.006
Female	14,589 (74.0)	45,105 (77.3)	0.077	13,661 (74.5)	13,751 (75.0)	0.011
White	9,976 (50.6)	41,683 (71.4)	0.437	9,865 (53.8)	9,847 (53.7)	0.002
Black or African American	4,569 (23.2)	6,260 (10.7)	0.336	3,742 (20.4)	3,731 (20.4)	0.001
Asian	875 (4.4)	2,961 (5.1)	0.030	852 (4.7)	830 (4.5)	0.006
Comorbidities and healthcare utilization						
Encounter for general examination	4,532 (23.0)	15,774 (27.0)	0.093	4,289 (23.4)	4,259 (23.2)	0.004
Essential (primary) hypertension	7,781 (39.5)	24,246 (41.5)	0.042	7,238 (39.5)	7,361 (40.1)	0.014
Dorsalgia	5,232 (26.5)	15,755 (27.0)	0.010	4,825 (26.3)	4,750 (25.9)	0.009
Other nutritional deficiencies	4,665 (23.7)	17,554 (30.1)	0.145	4,469 (24.4)	4665 (25.4)	0.025
Neoplasms	4,346 (22.0)	15,148 (26.0)	0.092	4,121 (22.5)	4,106 (22.4)	0.002
Disorders of thyroid gland	3,253 (16.5)	12,622 (21.6)	0.131	3,110 (17.0)	3,145 (17.2)	0.005
Diabetes mellitus	3,420 (17.3)	8,360 (14.3)	0.083	3,092 (16.9)	3,109 (17.0)	0.002
Chronic pain	3,282 (16.6)	9,520 (16.3)	0.009	3,008 (16.4)	3,016 (16.5)	0.001
Systemic connective tissue disorders	2,490 (12.6)	7,798 (13.4)	0.022	2,277 (12.4)	2,329 (12.7)	0.009
Sleep disorders	2174 (11.0)	6,292 (10.8)	0.008	2,024 (11.0)	2,028 (11.1)	0.001
Ischemic heart diseases	2070 (10.5)	6,394 (11.0)	0.015	1,916 (10.5)	1,943 (10.6)	0.005
Nicotine dependence	2,063 (10.5)	3,269 (5.6)	0.180	1,703 (9.3)	1,659 (9.1)	0.008
Anxiety disorders	1,720 (8.7)	4,552 (7.8)	0.034	1,559 (8.5)	1,621 (8.8)	0.012
Iron deficiency anemia	1,707 (8.7)	3,713 (6.4)	0.087	1,440 (7.9)	1,482 (8.1)	0.008
Diseases of liver	1,457 (7.4)	3,560 (6.1)	0.051	1,300 (7.1)	1,285 (7.0)	0.003
Chronic kidney disease (CKD)	1,406 (7.1)	4,339 (7.4)	0.012	1,293 (7.1)	1,270 (6.9)	0.005
COPD	1,323 (6.7)	3,353 (5.7)	0.040	1,202 (6.6)	1,241 (6.8)	0.009
Heart failure	1,221 (6.2)	2,903 (5.0)	0.053	1,058 (5.8)	1,093 (6.0)	0.008
Fibromyalgia	1,024 (5.2)	2,717 (4.7)	0.025	947 (5.2)	984 (5.4)	0.009
Cerebrovascular diseases	775 (3.9)	2,668 (4.6)	0.032	731 (4.0)	779 (4.3)	0.013
COVID-19	649 (3.3)	2,120 (3.6)	0.019	612 (3.3)	583 (3.2)	0.009
Conductive and sensorineural hearing loss	452 (2.3)	2,075 (3.6)	0.075	441 (2.4)	448 (2.4)	0.002
Malnutrition	419 (2.1)	710 (1.2)	0.071	328 (1.8)	367 (2.0)	0.016
Alcohol related disorders	401 (2.0)	543 (0.9)	0.091	299 (1.6)	288 (1.6)	0.005
Reduced mobility	175 (0.9)	399 (0.7)	0.023	151 (0.8)	161 (0.9)	0.006
Laboratory data						
Hemoglobin ≥ 12 g/dL	14,039 (71.2)	40,477 (69.3)	0.040	13,029 (71.1)	13,181 (71.9)	0.018
Albumin ≤ 3.5 g/dL	4,643 (23.5)	9,751 (16.7)	0.171	3,994 (21.8)	4,090 (22.3)	0.013
HbA1c ≥ 9%	739 (3.8)	1043 (1.8)	0.120	600 (3.3)	612 (3.3)	0.004
eGFR ≤ 60 mL/min/1.73 m^2^	3,429 (17.4)	10,815 (18.5)	0.030	3,178 (17.3)	3,263 (17.8)	0.012
C-reactive protein≥ 10 mg/L	5,460 (27.7)	10,913 (18.7)	0.214	4,779 (26.1)	4,861 (26.5)	0.010
Medications						
Corticosteroids for systemic use	10,741 (54.5)	30,539 (52.3)	0.043	9,844 (53.7)	9,894 (54.0)	0.005
Opioid analgesics	8,492 (43.1)	22,039 (37.8)	0.108	7,618 (41.5)	7,700 (42.0)	0.009
Benzodiazepine	4,746 (24.1)	13,548 (23.2)	0.020	4,296 (23.4)	4,377 (23.9)	0.010
Methotrexate	4,173 (21.2)	12,502 (21.4)	0.006	3,897 (21.3)	3,904 (21.3)	0.001
Hydroxychloroquine	3,344 (17.0)	10,119 (17.3)	0.010	3,117 (17.0)	3,092 (16.9)	0.004
Vitamin D supplementation	2,863 (14.5)	11,941 (20.5)	0.157	2,758 (15.0)	2,905 (15.8)	0.022
TNF-alpha inhibitors	2,599 (13.2)	6,765 (11.6)	0.048	2,386 (13.0)	2,385 (13.0)	0.000
Blood glucose-lowering drugs, excl. insulins	1,940 (9.8)	4,794 (8.2)	0.057	1,761 (9.6)	1,786 (9.7)	0.005
Insulins and analogs	1,768 (9.0)	3,368 (5.8)	0.123	1,510 (8.2)	1,571 (8.6)	0.012

Data are presented as *n* (%) or mean ± SD. SMD, standardized mean difference; BMI, body mass index; COPD, chronic obstructive pulmonary disease; CKD, chronic kidney disease; eGFR, estimated glomerular filtration rate; HbA1c, hemoglobin A1c; TNF, tumor necrosis factor; VDD, vitamin D deficiency. An SMD < 0.1 indicates adequate balance between groups.

### 3.2 Outcomes

**Figure 2:**
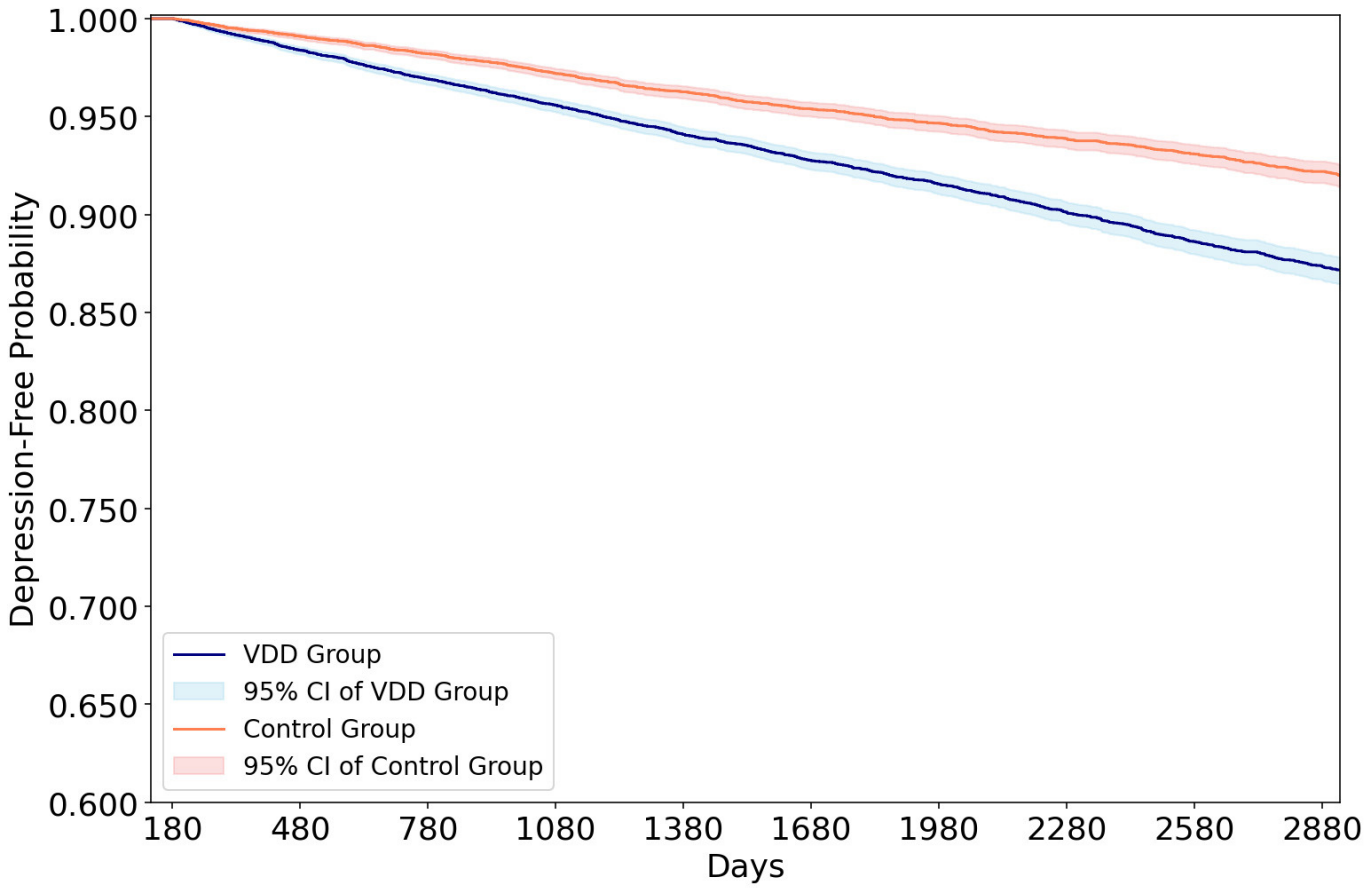
Kaplan–Meier curves for depression-free survival over 8 years in patients with rheumatoid arthritis, comparing the vitamin D deficiency group (< 20 ng/mL) with the control group (≥ 30 ng/mL). Follow-up began after a 6-month landmark period from the index date. The VDD group demonstrated significantly lower depression-free survival than the control group (log-rank *p* < 0.001). Shaded areas represent 95% confidence intervals. VDD, vitamin D deficiency; CI, confidence interval.

During follow-up, the mean follow-up time was 4.82 years in the VDD group and 4.72 years in the control group. Incident overall depression occurred in 1,390 patients (7.58%) in the VDD group and 847 patients (4.62%) in the control group, with an HR of 1.64 (95% CI, 1.50–1.78; *p* < 0.001) (Table 2). Kaplan–Meier analysis showed lower depression-free survival in the VDD group than in the control group throughout the follow-up (log-rank *p* < 0.001; Figure 2). When depression subtypes were analyzed separately, the VDD group was also associated with a higher risk of depressive episodes (HR 1.65, *p* < 0.001) and recurrent depressive disorder (HR 1.72, *p* < 0.001).

**Table 2:** Association between vitamin D deficiency and 8-year depression risk.

Outcome	VDD group (*n* = 18,339)	Control group (*n* = 18,339)	HR (95% CI)	*p* value
	Events (%)	Events (%)		
Primary outcome				
Overall depression	1,390 (7.58%)	847 (4.62%)	1.64 (1.50–1.78)	< 0.001
Depression episode	1,277 (6.96%)	772 (4.21%)	1.65 (1.51–1.80)	< 0.001
Recurrent depression	290 (1.58%)	165 (0.90%)	1.72 (1.42–2.08)	< 0.001
Secondary outcomes				
Suicide/self-harm^†^	45 (0.25%)	27 (0.15%)	1.62 (1.01–2.62)	0.045
Anti-depressant drug use	2,869 (15.64%)	1,999 (10.90%)	1.46 (1.38–1.55)	< 0.001
Mortality	1,601 (8.73%)	1,128 (6.15%)	1.40 (1.29–1.51)	< 0.001
Positive control outcome				
Osteoporotic fracture	242 (1.32%)	131 (0.71%)	1.80 (1.46–2.23)	< 0.001
Negative control outcome				
Acute appendicitis	53 (0.29%)	42 (0.23%)	1.24 (0.83–1.86)	0.297
Healthcare utilization validation				
Healthcare visit	17,282 (94.24%)	17,455 (95.18%)	0.93 (0.91–0.95)	< 0.001
Exposure validation				
Subsequent VDD diagnosis	5,126 (27.95%)	649 (3.54%)	9.45 (8.71–10.25)	< 0.001

Data are presented as *n* (%) for events and hazard ratios (HR) with 95% confidence intervals (CI). VDD, vitamin D deficiency.^†^Composite outcome of suicidal behavior, suicide attempt, and intentional self-harm.

For secondary outcomes, the VDD group was associated with a higher risk of antidepressant use (HR 1.46, *p* < 0.001), suicide-related outcomes (HR 1.62, *p* = 0.045), and all-cause mortality (HR 1.40, *p* < 0.001). As a positive control outcome, osteoporotic fracture was more frequently observed in the VDD group than in the control group (HR 1.80, *p* < 0.001). In contrast, no significant between-group difference was observed for the negative control outcome of acute appendicitis (HR 1.24, *p* = 0.297). During follow-up, healthcare visits were less frequent in the VDD group than in the control group (HR 0.93, *p* < 0.001). Subsequent VDD during follow-up was also more frequently observed among patients initially classified as vitamin D-deficient (HR 9.45, *p* < 0.001) (Table 2). The E-value for the primary outcome was 2.66 for the point estimate and 2.37 for the lower bound of the 95% confidence interval.

### 3.3 Sensitivity analyses

**Figure 3:**
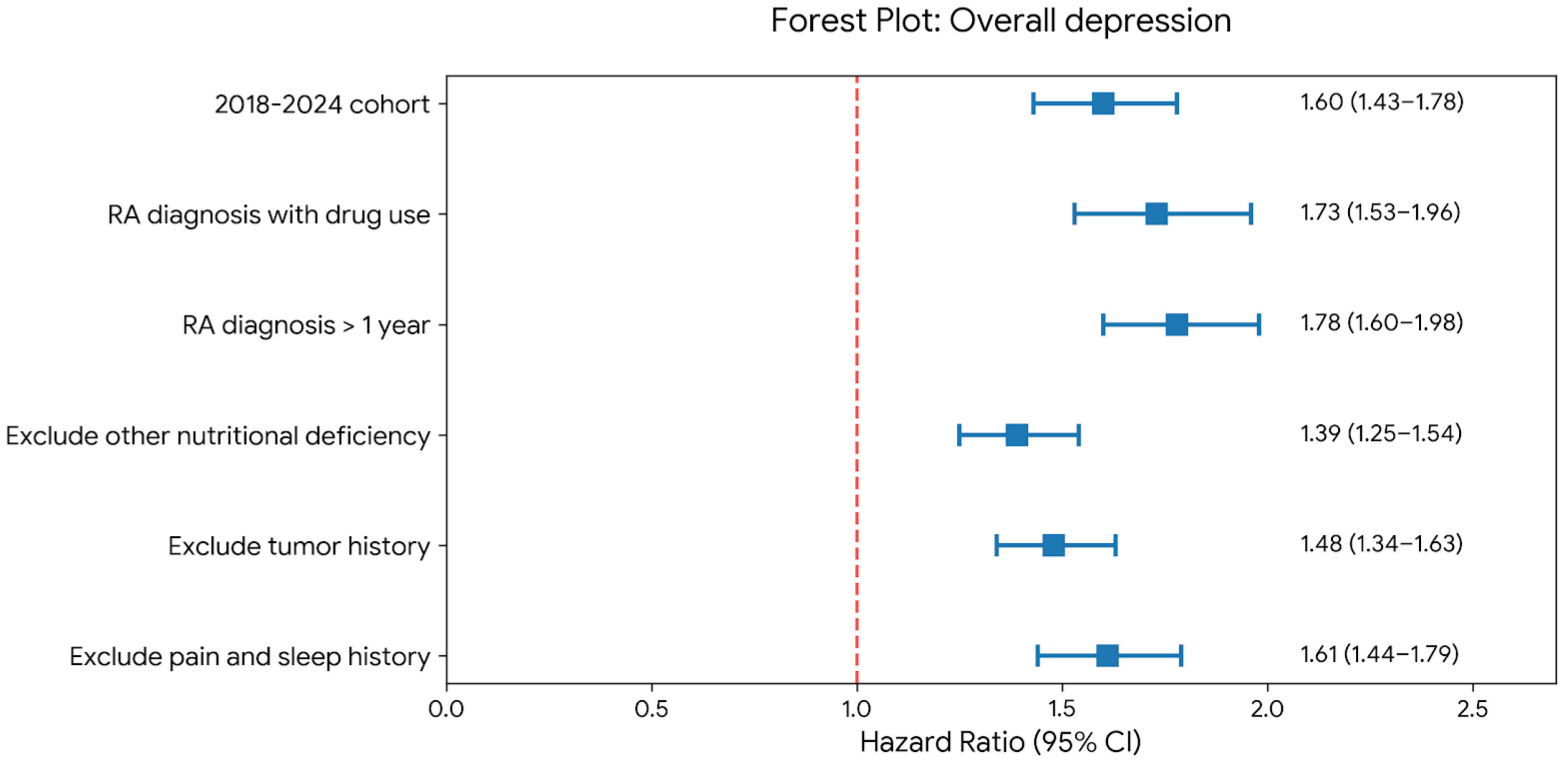
Forest plot of sensitivity analyses for the association between vitamin D deficiency and incident overall depression. Each analysis applied the same propensity score matching strategy and Cox proportional hazards regression as the primary analysis. The dashed vertical line indicates a hazard ratio of 1.0 (no association). HR, hazard ratio; CI, confidence interval.

An association between VDD and incident depression was observed across all prespecified sensitivity analyses (Figure 3). Similar results were obtained when the analysis was restricted to a more contemporary cohort (2018–2024), to patients receiving RA-related drug treatment, and to patients with a history of RA of more than 1 year. Similar findings were also observed after excluding patients with other nutritional deficiencies, a history of neoplasms, chronic pain, or sleep disorders. Across these analyses, the HRs remained in the same direction and were statistically significant.

### 3.4 Subgroup analyses

In subgroup analyses according to sex, an association between VDD and depression was observed in both males (HR 1.76, *p* < 0.001) and females (HR 1.68, *p* < 0.001); however, the interaction by sex was not significant (*p* for interaction = 0.669). Similar patterns were observed for depressive episodes, antidepressant use, and all-cause mortality in both subgroups. For recurrent depression, the association was statistically significant in women (HR 1.55, *p* < 0.001) but not in men (HR 1.53, *p* = 0.052). Suicide-related outcomes could not be reliably evaluated in men because of the limited number of events (Table 3).

**Table 3:** Subgroup analyses of the association between vitamin D deficiency and 8-year depression risk, stratified by sex.

Outcomes	Male (*n* = 4,638)		Female (*n* = 13,677)		*P* for interaction
	HR (95% CI)	*P* value	HR (95% CI)	*P* value	
Overall depression	1.76 (1.46–2.12)	< 0.001	1.68 (1.53–1.85)	< 0.001	0.669
Depression episode	1.80 (1.48–2.19)	< 0.001	1.75 (1.58–1.93)	< 0.001	0.804
Recurrent depression	1.53 (0.99–2.36)	0.052	1.55 (1.26–1.90)	< 0.001	0.959
Suicide/self-harm^†^	–	–	1.77 (0.96–3.26)	0.064	-
Anti-depressant drug use	1.35 (1.20–1.52)	< 0.001	1.44 (1.35–1.54)	< 0.001	0.343
Mortality	1.33 (1.17–1.52)	< 0.001	1.41 (1.29–1.55)	< 0.001	0.472

Data are presented as hazard ratios (HR) with 95% confidence intervals (CI). Suicide/self-harm results in males were not estimable due to insufficient events.^†^Composite outcome of suicidal behavior, suicide attempt, and intentional self-harm.

### 3.5 Adjusted hazard ratios for depression from multivariable Cox regression

In the multivariate Cox regression model, VDD was associated with incident depression after adjusting for demographic and clinical covariates (HR 1.71, *p* < 0.001). Other variables associated with a higher risk of incident depression included anxiety disorders (HR 1.95, *p* < 0.001), nicotine dependence (HR 1.59, *p* < 0.001), pain (HR 1.55, *p* < 0.001), sleep disorders (HR 1.42, *p* < 0.001), malnutrition (HR 1.34, *p* = 0.019), diabetes mellitus (HR 1.23, *p* < 0.001), and hypertension (HR 1.20, *p* < 0.001). Male sex was associated with a lower risk of incident depression (HR 0.83, *p* < 0.001). Age, other nutritional deficiencies, thyroid disorders, systemic connective tissue disorders, and chronic kidney disease were not significantly associated with incident depression (Table 4).

**Table 4:** Adjusted hazard ratios for incident depression from multivariate Cox proportional hazards regression.

Variable	Hazard ratio (95% CI)	*P*-value
VDD vs. control group	1.71 (1.60–1.83)	< 0.001
Male	0.83 (0.76–0.89)	< 0.001
Age at Index	1.00 (1.00–1.00)	0.093
Other nutritional deficiencies	0.97 (0.90–1.04)	0.380
Neoplasms	0.84 (0.78–0.91)	< 0.001
Disorders of thyroid gland	1.06 (0.98–1.14)	0.167
Diabetes mellitus	1.23 (1.13–1.34)	< 0.001
Essential (primary) hypertension	1.20 (1.12–1.29)	< 0.001
Pain, not elsewhere classified	1.55 (1.43–1.67)	< 0.001
Systemic connective tissue disorders	1.07 (0.98–1.17)	0.130
Sleep disorders	1.42 (1.30–1.55)	< 0.001
Nicotine dependence	1.59 (1.44–1.76)	< 0.001
Anxiety disorders	1.95 (1.79–2.14)	< 0.001
Chronic kidney disease (CKD)	1.07 (0.95–1.21)	0.289
Malnutrition	1.34 (1.05–1.71)	0.019

Data are presented as hazard ratios (HR) with 95% confidence intervals (CI). VDD, vitamin D deficiency; CKD, chronic kidney disease.

### 3.6 Exposure-gradient analysis

In the exposure–gradient analysis, baseline characteristics between the vitamin D insufficiency and control groups were well balanced after matching, with all standardized mean differences < 0.1 (Supplementary Table 5). Compared with the sufficient vitamin D group, vitamin D insufficiency (20–29.9 ng/mL) was associated with a higher risk of overall depression (HR 1.46, *p* < 0.001), depressive episode (HR 1.47, *p* < 0.001), recurrent depression (HR 1.31, *p* = 0.001), suicide-related outcomes (HR 1.53, *p* = 0.036), antidepressant use (HR 1.34, *p* < 0.001), and all-cause mortality (HR 1.10, *p* = 0.004). These effect estimates were smaller than those observed in the primary analysis of VDD vs. sufficient vitamin D levels (Table 5).

**Table 5:** Association between vitamin D insufficiency (20–29.9 ng/mL) and 8-year risk of depression and secondary outcomes compared with sufficient vitamin D levels (≥30 ng/mL).

Outcome	VDI group (*n* = 24,916)	Control group (*n* = 24,916)	HR (95% CI)	*p* value
	Events (%)	Events (%)		
Overall depression	1,764 (7.08%)	1,196 (4.80%)	1.46 (1.35–1.57)	< 0.001
Depression episode	1,616 (6.49%)	1,080 (4.34%)	1.47 (1.36–1.59)	< 0.001
Recurrent depression	340 (1.37%)	251 (1.01%)	1.31 (1.11–1.54)	0.001
Suicide/self-harm^†^	62 (0.25%)	39 (0.16%)	1.53 (1.03–2.29)	0.036
Anti-depressant drug use	3,841 (15.42%)	2,870 (11.52%)	1.34 (1.28–1.41)	< 0.001
Mortality	1,887 (7.57%)	1,659 (6.66%)	1.10 (1.03–1.18)	0.004

Data are presented as *n* (%) for events and hazard ratios (HR) with 95% confidence intervals (CI). VDI, vitamin D insufficiency.^†^Composite outcome of suicidal behavior, suicide attempt, and intentional self-harm.

## 4 Discussion

In this large-scale propensity score-matched cohort of patients with RA, VDD was associated with a significantly higher 8-year risk of incident depression. This association remained consistent across all prespecified sensitivity analyses and persisted after multivariate adjustment for demographic and clinical covariates. An exposure gradient pattern was also observed, with vitamin D insufficiency showing a similar but smaller effect estimate. The credibility of these findings was further supported by the expected positive control result, the null finding for the negative control outcome, and the fact that healthcare visits during follow-up were slightly less frequent in the VDD group than in the control group, which does not support increased healthcare utilization as an explanation for the higher observed depression risk. However, secondary and exploratory outcomes should be interpreted with caution given the absence of adjustment for multiple comparisons, and findings with marginal significance may represent chance findings.

To our knowledge, this is the first longitudinal study to examine the temporal association between VDD and incident depression specifically in the RA population. Prior evidence on this topic has been limited to cross-sectional data. Pu et al. (13) reported that among 161 patients with RA, those with depression had significantly lower serum vitamin D levels than those without depression, and that vitamin D was inversely correlated with Hamilton Depression Scale scores. However, the cross-sectional design of that study precluded the determination of temporal directionality and could not distinguish whether low vitamin D levels preceded depression or whether depression-related behavioral changes, such as reduced outdoor activity and poor dietary intake, led to vitamin D depletion. By employing a landmark analysis design with an 180-day washout period and excluding patients with prevalent depression or antidepressant use at baseline, the present study strengthens the temporal inference that VDD precedes the onset of depression. Furthermore, our sample size was substantially larger, allowing for more precise effect estimates and comprehensive subgroup and sensitivity analyses. A recent narrative review on RA-associated depression identified VDD as a potential risk factor warranting longitudinal investigation (14), and the present study directly addresses this evidence gap.

Several existing studies have identified disease activity, pain, functional disability, and corticosteroid exposure as risk factors for depression in RA (15, 16). Our multivariable Cox regression confirmed that pain, anxiety disorders, sleep disorders, and nicotine dependence were independently associated with incident depression. Notably, VDD retained an independent association with depression after adjustment for these established risk factors, with a hazard ratio of 1.71, suggesting that vitamin D status may be an additional marker associated with depression risk in RA, rather than implying a causal or modifiable effect. In current study, the suicide/self-harm analysis should also be interpreted cautiously because of the small number of events. Although the overall association reached nominal statistical significance, the estimate was based on few events and had a wide confidence interval; moreover, sex-stratified analyses were not estimable in men and were not statistically significant in women. Because TriNetX does not support exact Poisson regression or Firth penalized likelihood models, more stable rare-event modeling could not be performed.

Several biological mechanisms may underlie the observed association between VDD and depression in patients with RA. First, vitamin D has been shown to transcriptionally activate tryptophan hydroxylase 2 (TPH2), the rate-limiting enzyme for serotonin synthesis in the brain (7, 17). In patients with RA, chronic systemic inflammation preferentially drives tryptophan metabolism toward the pathway, thereby reducing the substrate availability for serotonin production (18). Concurrent VDD may compound this effect by further downregulating TPH2 expression, thereby creating a dual impairment of central serotonin synthesis (19). Second, vitamin D exerts immunomodulatory effects by suppressing pro-inflammatory cytokines including TNF-α and IL-6 (20–22), both of which are characteristically elevated in RA (23–26), and have been implicated in the cytokine hypothesis of depression (27–29). VDD in the context of RA may therefore amplify neuroinflammatory signaling, facilitating the development of depressive symptoms. Third, dysregulation of the hypothalamic–pituitary–adrenal (HPA) axis has been proposed as a shared pathway linking VDD and depression (6, 30, 31). In patients with RA, this pathway may be further perturbed by corticosteroid use, which was documented in over half of the study population (32–34).

Exposure–gradient analysis revealed a graded association between vitamin D status and depression risk, with VDD conferring a higher risk than insufficiency (35–38). This dose–response pattern is consistent with the Bradford Hill criterion of biological gradient and suggests that the association is unlikely to be entirely attributable to residual confounding (39, 40). From a clinical perspective, these findings indicate that even vitamin D levels in the insufficiency range (20–29.9 ng/mL) were associated with an elevated depression risk compared with levels ≥30 ng/mL (41), although this observation reflects an association and should not be interpreted as causal. This observation aligns with the Endocrine Society guideline recommendation of maintaining serum 25-hydroxyvitamin D levels at or above 30 ng/mL (42) and suggests that this threshold may be relevant not only for musculoskeletal health but also for mental health in the RA population. Subgroup analyses demonstrated that the association between VDD and depression was consistent across both sexes, with no significant interaction effect. This finding has practical implications, as depression in men with RA is frequently underrecognized (43), and vitamin D screening could serve as a sex-neutral strategy for identifying patients at elevated risk (44–46).

The study design incorporated several features to address common sources of bias in observational research. The 180-day landmark period reduced the likelihood of reverse causation and misclassification of prevalent depression as an incident disease. During follow-up, healthcare visits were slightly less frequent in the VDD group than in the control group, which does not support greater surveillance as an explanation for the higher observed depression risk. Exposure classification was further strengthened by requiring stable vitamin D status over the preceding three years, and this approach was supported by the markedly higher rate of subsequent VDD among patients initially classified as deficient. In addition, the E-value of 2.66 suggests that an unmeasured confounder would need to have a moderately strong association with both VDD and depression to fully account for the observed association, beyond the measured covariates. The null finding for the negative control outcome reduces concern about substantial systematic bias, whereas the expected association for osteoporotic fractures supports the validity of the exposure classification.

Several limitations merit consideration. First, the TriNetX database does not capture RA disease activity indices (e.g., DAS28, CDAI) or functional status, which are important potential confounders of the vitamin D–depression relationship (47). Although RA-related medications and C-reactive protein were included as proxies, residual confounding from disease activity may persist (48). Because higher disease activity is plausibly associated with both lower vitamin D levels and increased depression risk, the observed association may be overestimated. In addition, lifestyle factors such as outdoor activity and sunlight exposure, which influence vitamin D status and may independently affect depression risk, were not available in the database. Second, vitamin D status was assessed using a single baseline measurement, and within-person variability related to seasonal changes, supplementation, and changes in health status may have introduced exposure misclassification (49, 50). Although this concern was partially mitigated by excluding patients with discordant prior vitamin D measurements and by the markedly higher rate of subsequent VDD in the deficient group, regression dilution bias may still have occurred and would likely attenuate the observed association. Third, depression was identified using ICD diagnostic codes rather than standardized instruments such as the PHQ-9 or structured clinical interviews. This approach may have led to under-ascertainment of mild or subthreshold depression and potential diagnostic misclassification. However, such misclassification would likely be non-differential between groups and therefore bias the results toward the null. Fourth, the TriNetX network predominantly comprises healthcare organizations in the United States, and variations in vitamin D status, supplementation practices, sunlight exposure, and RA management across regions may limit extrapolation to other populations. In addition, although race was balanced after matching, the cohort remained predominantly White with a relatively small proportion of Asian patients, and race-stratified analyses were not performed. Therefore, potential heterogeneity in the association between VDD and depression across racial or ethnic groups cannot be excluded. Finally, residual reverse causation cannot be fully excluded. Although a 180-day landmark design was applied and patients with prior depression or antidepressant use were excluded, subclinical depressive symptoms preceding formal diagnosis may have influenced health behaviors, such as reduced outdoor activity and sunlight exposure, leading to lower vitamin D levels before the landmark period. Because such early, undiagnosed symptoms are not captured in the database, the temporal direction between VDD and depression may be partially obscured. In addition, several laboratory variables exhibited partial missingness, and analyses were conducted using available data without imputation. Although the proportion of available laboratory data was similar between groups after matching, the pattern of missingness (e.g., whether missingness is related to exposure or outcome) could not be formally evaluated. Furthermore, the TriNetX platform does not support multiple imputation or inverse probability weighting, precluding formal sensitivity analyses to address potential selection bias. Therefore, the possibility of bias related to non-random missingness and related selection bias cannot be excluded.

In conclusion, this study provides the first large-scale longitudinal evidence that VDD is associated with an increased risk of incident depression in patients with RA. This association was independent of established risk factors, exhibited a dose–response pattern, and was supported by multiple methodological safeguards against bias. These findings suggest that VDD is associated with an increased risk of incident depression in patients with RA. However, given the observational nature of this study, these findings should not be interpreted as evidence of a causal or modifiable effect, and further studies are needed to clarify whether this association is causal.

## Data Availability

The original contributions presented in the study are included in the article/Supplementary material, further inquiries can be directed to the corresponding author.
