# Supplementary material for: Association between vitamin D deficiency and the 8-year risk of incident depression in patients with rheumatoid arthritis: a cohort study
Source: Front Nutr. 2026 May 21;13:1845922. doi: 10.3389/fnut.2026.1845922 (PMC13233263; doi:10.3389/fnut.2026.1845922)
Supplement: Supplementary file 1 [file Table_1.docx]

**Supplemental Table 1. Description of the TriNetX Global Network**

| The TriNetX Global Network (https://live.trinetx.com) is a federated health research network that provides access to de-identified electronic health record (EHR) data from participating healthcare organizations. The network includes hospitals, academic medical centers, and specialty care providers, primarily in the United States. The database contains patient-level information including demographics, diagnoses coded using the International Classification of Diseases, Tenth Revision, Clinical Modification (ICD-10-CM), procedures coded using CPT and ICD procedure codes, medication prescriptions, laboratory test results, and healthcare utilization data.  TriNetX operates under a federated data model in which participating institutions retain physical control of their data, and only aggregated statistical results are returned to users. No direct patient identifiers are available to researchers. The platform includes built-in analytics tools for cohort selection, propensity score matching, survival analysis, and risk estimation. The TriNetX network has been widely used in observational cohort studies across multiple clinical disciplines, including epidemiology, oncology, cardiovascular medicine, and outcomes research. |
| --- |

**Supplemental Table 2. Codes Used for Cohort Definition, Inclusion Criteria, Exclusion Criteria, and Outcome Definitions**

| Category | Variable / Definition | Codes / Terms Used |
| --- | --- | --- |
| Cohort definition | Vitamin D testing | TNX:9034 Calcidiol [Mass/volume] in Serum or Plasma; TNX:LG25965-1 Calcidiol + ercalcidiol |
|  | Vitamin D deficiency | Calcidiol ≤19.9 ng/mL |
|  | Control group (Vitamin D sufficient) | Calcidiol ≥30.0 ng/mL |
|  | Rheumatoid arthritis | ICD-10: M05 (Rheumatoid arthritis with rheumatoid factor), M06 (Other rheumatoid arthritis) |
|  | Age | ≥18 years |
| Inclusion criteria | Serum 25-hydroxyvitamin D measurement | TNX:9034, TNX:LG25965-1 |
|  | Rheumatoid arthritis diagnosis | ICD-10: M05, M06 |
|  | Study period | Jan 1, 2010 – Dec 31, 2024 |
| Exclusion criteria | Prior depression | ICD-10: F32, F33 |
|  | Suicidal ideation | ICD-10: R45.851 |
|  | Suicide attempt | ICD-10: T14.91 |
|  | Intentional self-harm | ICD-10: X71–X83 |
|  | Antidepressant use | ATC: N06A |
|  | End-stage renal disease | ICD-10: N18.6 |
|  | Advanced chronic kidney disease | ICD-10: N18.4, N18.5 |
|  | Dialysis dependence | ICD-10: Z99.2 |
|  | Bipolar disorder | ICD-10: F31 |
|  | Schizophrenia and psychotic disorders | ICD-10: F20–F29 |
|  | Bariatric surgery | ICD-10: Z98.84 |
|  | Bariatric surgery procedures | CPT: 43775, 43644, 1007385, 1007386 |
|  | Stroke | ICD-10: I63 |
|  | Intracerebral hemorrhage | ICD-10: I61 |
| Outcome definitions | Depressive episode | ICD-10: F32 |
|  | Recurrent depression | ICD-10: F33 |
|  | Overall depression | ICD-10: F32, F33 |
|  | Suicide outcomes | ICD-10: R45.851, T14.91, X71–X83 |
|  | Osteoporosis with fracture | ICD-10: M80 |
|  | Appendicitis (negative control) | ICD-10: K35 |
|  | Mortality | Deceased status; ICD-10: R99 |
|  | Antidepressant use | ATC: N06A |
|  | Vitamin D deficiency during follow-up | TNX:9034, TNX:LG25965-1 (≤19.9 ng/mL) |

**Supplemental Table 3. Medications Used to Confirm the Diagnosis of Rheumatoid Arthritis**

| Category | Medication | RxNorm Code |
| --- | --- | --- |
| Conventional DMARD | Methotrexate | 6851 |
| Conventional DMARD | Sulfasalazine | 9524 |
| Conventional DMARD | Hydroxychloroquine | 5521 |
| Conventional DMARD | Leflunomide | 27169 |
| TNF inhibitor | Adalimumab | 327361 |
| TNF inhibitor | Etanercept | 214555 |
| TNF inhibitor | Infliximab | 191831 |
| TNF inhibitor | Golimumab | 819300 |
| TNF inhibitor | Certolizumab | OMOP5175190 |
| IL-6 inhibitor | Tocilizumab | 612865 |
| IL-6 inhibitor | Sarilumab | 1923319 |
| T-cell costimulation inhibitor | Abatacept | 614391 |
| B-cell depletion agent | Rituximab | 121191 |
| JAK inhibitor | Tofacitinib | 1357536 |
| JAK inhibitor | Baricitinib | 2047232 |
| JAK inhibitor | Upadacitinib | 2196092 |
| JAK inhibitor | Filgotinib | OMOP5036197 |

**Supplemental Table 4. Availability of laboratory data Before and After Propensity Score Matching**

| Variable | Before Matching VDD n (%) | Before Matching Control n (%) | After Matching VDD n (%) | After Matching Control n (%) |
| --- | --- | --- | --- | --- |
| Hemoglobin | 16,075 (81.49%) | 44,504 (76.23%) | 14,834 (80.89%) | 14,646 (79.86%) |
| eGFR (CKD-EPI) | 15,410 (78.12%) | 43,615 (74.70%) | 14,296 (77.95%) | 13,884 (75.71%) |
| Albumin | 14,368 (72.84%) | 40,996 (70.22%) | 13,220 (72.09%) | 13,284 (72.44%) |
| BMI | 13,475 (68.31%) | 40,220 (68.89%) | 12,346 (67.32%) | 13,091 (71.38%) |
| C-reactive protein | 9,735 (49.35%) | 23,465 (40.19%) | 8,864 (48.33%) | 8,564 (46.70%) |
| HbA1c | 6,111 (30.98%) | 16,335 (27.98%) | 5,583 (30.44%) | 5,511 (30.05%) |

**Supplemental Table 5.** **Baseline characteristics of patients with vitamin D insufficiency and controls before and after propensity score matching**

| Variables | Before matching | | | After matching | | | |
| --- | --- | --- | --- | --- | --- | --- | --- |
|  | VDD group  (n = 25,550) | Control group  (n = 49,859) | SMD | | VDD group  (n = 24,916) | Control group  (n = 24,916) | SMD |
| Patient characteristics |  |  |  | |  |  |  |
| Age at index (years) | 57.3±16.0 | 63.1±14.5 | 0.380 | | 57.9±15.6 | 58.0±15.4 | 0.004 |
| BMI ≥30 (kg/m^2^) | 9264 (36.3) | 15107 (30.3) | 0.127 | | 8940 (35.9) | 8946 (35.9) | 0.001 |
| Female | 18924 (74.1) | 38630 (77.5) | 0.080 | | 18522 (74.3) | 18414 (73.9) | 0.010 |
| White | 15578 (61.0) | 35395 (71.0) | 0.213 | | 15439 (62.0) | 15410 (61.8) | 0.002 |
| Black or African American | 3947 (15.4) | 5739 (11.5) | 0.115 | | 3772 (15.1) | 3727 (15.0) | 0.005 |
| Asian | 1295 (5.1) | 2358 (4.7) | 0.016 | | 1269 (5.1) | 1236 (5.0) | 0.006 |
| Comorbidities and Healthcare Utilization | | |  | |  |  |  |
| Encounter for general examination | 6338 (24.8) | 13183 (26.4) | 0.037 | | 6225 (25.0) | 6243 (25.1) | 0.002 |
| Essential (primary) hypertension | 9928 (38.9) | 20772 (41.7) | 0.057 | | 9769 (39.2) | 9756 (39.2) | 0.001 |
| Other nutritional deficiencies | 7166 (28.0) | 13901 (27.9) | 0.004 | | 6979 (28.0) | 6967 (28.0) | 0.001 |
| Dorsalgia | 7109 (27.8) | 13328 (26.7) | 0.025 | | 6919 (27.8) | 6917 (27.8) | 0.000 |
| Neoplasms | 5959 (23.3) | 12785 (25.6) | 0.054 | | 5888 (23.6) | 5779 (23.2) | 0.010 |
| Disorders of thyroid gland | 4497 (17.6) | 10629 (21.3) | 0.094 | | 4456 (17.9) | 4391 (17.6) | 0.007 |
| Pain, not elsewhere classified | 4349 (17.0) | 8349 (16.7) | 0.007 | | 4231 (17.0) | 4273 (17.2) | 0.004 |
| Diabetes mellitus | 3987 (15.6) | 7244 (14.5) | 0.030 | | 3887 (15.6) | 3936 (15.8) | 0.005 |
| Systemic connective tissue disorders | 3326 (13.0) | 6657 (13.4) | 0.010 | | 3242 (13.0) | 3309 (13.3) | 0.008 |
| Sleep disorders | 2845 (11.1) | 5374 (10.8) | 0.011 | | 2787 (11.2) | 2788 (11.2) | 0.000 |
| Ischemic heart diseases | 2622 (10.3) | 5598 (11.2) | 0.031 | | 2587 (10.4) | 2577 (10.3) | 0.001 |
| Anxiety, dissociative, stress-related, somatoform and other nonpsychotic mental disorders | 2197 (8.6) | 3881 (7.8) | 0.030 | | 2122 (8.5) | 2121 (8.5) | 0.000 |
| Nicotine dependence | 2032 (8.0) | 2972 (6.0) | 0.078 | | 1950 (7.8) | 1921 (7.7) | 0.004 |
| Iron deficiency anemia | 1917 (7.5) | 3207 (6.4) | 0.042 | | 1816 (7.3) | 1807 (7.3) | 0.001 |
| Diseases of liver | 1698 (6.6) | 3067 (6.2) | 0.020 | | 1656 (6.6) | 1682 (6.8) | 0.004 |
| Chronic kidney disease (CKD) | 1609 (6.3) | 3846 (7.7) | 0.056 | | 1593 (6.4) | 1576 (6.3) | 0.003 |
| Other chronic obstructive pulmonary disease | 1460 (5.7) | 3007 (6.0) | 0.013 | | 1442 (5.8) | 1503 (6.0) | 0.010 |
| Fibromyalgia | 1451 (5.7) | 2251 (4.5) | 0.053 | | 1391 (5.6) | 1356 (5.4) | 0.006 |
| Heart failure | 1247 (4.9) | 2650 (5.3) | 0.020 | | 1224 (4.9) | 1213 (4.9) | 0.002 |
| Cerebrovascular diseases | 1025 (4.0) | 2309 (4.6) | 0.030 | | 1011 (4.1) | 1004 (4.0) | 0.001 |
| COVID-19 | 867 (3.4) | 1878 (3.8) | 0.020 | | 846 (3.4) | 859 (3.4) | 0.003 |
| Conductive and sensorineural hearing loss | 708 (2.8) | 1791 (3.6) | 0.047 | | 703 (2.8) | 711 (2.9) | 0.002 |
| Malnutrition | 417 (1.6) | 663 (1.3) | 0.025 | | 394 (1.6) | 376 (1.5) | 0.006 |
| Alcohol related disorders | 311 (1.2) | 539 (1.1) | 0.013 | | 299 (1.2) | 319 (1.3) | 0.007 |
| Reduced mobility | 185 (0.7) | 373 (0.7) | 0.003 | | 179 (0.7) | 181 (0.7) | 0.001 |
| Laboratory data |  |  |  | |  |  |  |
| Hemoglobin ≥ 12 g/dL | 18425 (72.1) | 33919 (68.0) | 0.089 | | 17884 (71.8) | 17937 (72.0) | 0.005 |
| Albumin ≤3.5 g/dL) | 4999 (19.6) | 8588 (17.2) | 0.060 | | 4787 (19.2) | 4755 (19.1) | 0.003 |
| HbA1c ≥ 9% | 708 (2.8) | 933 (1.9) | 0.060 | | 659 (2.6) | 664 (2.7) | 0.001 |
| eGFR ≤ 60 mL/min/1.73 m² | 4043 (15.8) | 9536 (19.1) | 0.087 | | 4004 (16.1) | 4019 (16.1) | 0.002 |
| C-reactive protein≥ 10 mg/L | 6300 (24.7) | 9462 (19.0) | 0.138 | | 5957 (23.9) | 5950 (23.9) | 0.001 |
| Medications |  |  |  | |  |  |  |
| Corticosteroids for systemic use | 14146 (55.4) | 26164 (52.5) | 0.058 | | 13724 (55.1) | 13703 (55.0) | 0.002 |
| Opioid analgesics | 10380 (40.6) | 18841 (37.8) | 0.058 | | 10065 (40.4) | 9957 (40.0) | 0.009 |
| Benzodiazepine | 6028 (23.6) | 11636 (23.3) | 0.006 | | 5876 (23.6) | 5771 (23.2) | 0.010 |
| Methotrexate | 6003 (23.5) | 10786 (21.6) | 0.045 | | 5785 (23.2) | 5816 (23.3) | 0.003 |
| Hydroxychloroquine | 4600 (18.0) | 8705 (17.5) | 0.014 | | 4486 (18.0) | 4459 (17.9) | 0.003 |
| Vitamin d supplementation | 5213 (20.4) | 10165 (20.4) | 0.000 | | 5070 (20.3) | 5038 (20.2) | 0.003 |
| TNF-alpha inhibitors | 3476 (13.6) | 5719 (11.5) | 0.064 | | 3322 (13.3) | 3313 (13.3) | 0.001 |
| Blood glucose-lowering drugs, excl. Insulins | 2338 (9.2) | 4144 (8.3) | 0.030 | | 2277 (9.1) | 2281 (9.2) | 0.001 |
| Insulins and analogues | 1785 (7.0) | 3030 (6.1) | 0.037 | | 1724 (6.9) | 1737 (7.0) | 0.002 |

Data are presented as n (%) or mean ± SD. SMD, standardized mean difference; BMI, body mass index; COPD, chronic obstructive pulmonary disease; CKD, chronic kidney disease; eGFR, estimated glomerular filtration rate; HbA1c, hemoglobin A1c; TNF, tumor necrosis factor; VDD, vitamin D deficiency. An SMD < 0.1 indicates adequate balance between groups.
